# Implementation of a Diabetes Educator Care Model to Reduce Paediatric Admission for Diabetic Ketoacidosis

**DOI:** 10.1155/2016/3917806

**Published:** 2016-05-17

**Authors:** Asma Deeb, Hana Yousef, Layla Abdelrahman, Mary Tomy, Shaker Suliman, Salima Attia, Hana Al Suwaidi

**Affiliations:** Paediatric Endocrinology Department, Mafraq Hospital, P.O. Box 2951, Abu Dhabi, UAE

## Abstract

*Introduction*. Diabetic Ketoacidosis (DKA) is a serious complication that can be life-threatening. Management of DKA needs admission in a specialized center and imposes major constraints on hospital resources.* Aim*. We plan to study the impact of adapting a diabetes-educator care model on reducing the frequency of hospital admission of children and adolescents presenting with DKA.* Method*. We have proposed a model of care led by diabetes educators for children and adolescents with diabetes. The team consisted of highly trained nurses. The model effectiveness is measured by comparing the rate of hospital admission for DKA over 4-year period to the baseline year prior to implementing the model.* Results*. There were 158 admissions for DKA over a 5-year period. Number of patients followed up in the outpatient diabetes clinics increased from 37 to 331 patients at the start and the end of the study years. Admission rate showed a downward trend over the five-year period. Percentage of admission for DKA is reduced from 210% to 1.8% (*P* 0.001).* Conclusion*. Diabetes educator care model is an effective and a sustainable measure to reduce hospital admission for DKA in children and adolescents.

## 1. Introduction

Diabetic Ketoacidosis (DKA) is a common but a preventable complication. It is associated with a significant potential for long-term morbidity and mortality. It is estimated that the risk of DKA in established type 1 diabetes patients can be up to 10% per patient per year [1]. In national population studies, the mortality rate from DKA in children is 0.15% to 0.30% [2]. Cerebral edema is a known complication of DKA and it accounts for 60% to 90% of all DKA deaths [3]. Of the cerebral oedema survivors, 10% to 25% have significant residual morbidity [2, 3].

It is known that health care costs for type 1 diabetes patients are higher than those for general population [4]. In particular, DKA is an expensive complication of type 1 diabetes. It is estimated that the mean cost per hospitalization is $10, 876 ± 11,024 [5]. DKA was listed as the primary diagnosis for 89,000 hospital admissions and as the secondary diagnosis for 113,000 admissions [6]. In another study, it is found that the average cost for DKA hospital admission of a person (paediatric or adult) is $13,000. This estimated cost results in a total cost of over $1 billion annually in the United States [7].

Lack of proper management and follow-up put the children at risk of DKA and other acute complications which can be life threatening. Poor follow-up and lack of access to service increase rate of hospital admission to treat acute complications. Lack of continuity of care and point of contact after hours are important factors in increasing hospital admission to treat DKA in children and adolescents. Diabetes control relies extensively on the caregivers' day to day care. Provision of open clinic access and after hours-point of contact is crucial [8]. Instant troubleshooting is required often for children with diabetes out of hours. Such service is an important aid to prevent and to treat DKA early. Immediate and directed instructions for care giver of a child with early signs of DKA can prevent its progression and avoid the need for hospital presentation and admission.

DKA results from deficiency of circulating insulin and the combined effects of increased levels of the counterregulatory hormones: catecholamines, glucagon, cortisol, and growth hormone [9]. It is not an uncommon complication in children and adolescents with established diabetes. Its severity can vary from mild acidosis to a life threatening condition. Mild DKA can progress to severe form if not managed properly. Accordingly, education of early signs of DKA and its management is of paramount importance to prevent and manage the condition.

Despite the improvement in diabetes management over the last decades, the frequency of DKA remained high [10]. Specialized diabetes nurse and diabetes educators are crucial members of the diabetes team. Their input is fundamental in establishing a center of excellence for quality management of diabetes in young people [11]. Training nurses in the diabetes team to be competent trainers should take a priority when setting up a diabetes center. Initial training should be followed by regular updating which requires commitment by the trainees and the employers [12].

Patient and family education is a major step in coping with diabetes management. It is proven that ensuring high family education and competence in the disease management is associated with better control, reduction of acute complication, and improvement of emotional well-being [13]. Patients should have access to a 24-hour telephone helpline managed by highly trained educators for emergency advice and treatment of DKA [14].

Experienced diabetes educators are valuable members of the diabetes team. We hypothesize that implementing a diabetes educator model of care for children and adolescents with diabetes reduces the frequency of hospital admission for DKA.

## 2. Materials and Methods

Information on the episodes on hospital admission for acute presentation of DKA was searched for. Data was obtained through Mafraq Hospital Health Information System (HIS) from the Medical Records Department. Search was limited to paediatric wards, medical wards, and intensive care units. Obtained data were filtered to capture number of admissions for patients 18 years old and under who have established diabetes and were admitted with the diagnosis of DKA. Venous blood pH was available in all patients included and reading below 7.3 was considered acidosis. Number and details of patients admitted were provided by the HIS Department. Data requested was for admission in the year of 2009 and 2010 (data point* A*). Patients who were admitted with DKA as a first sign of new diagnosis of diabetes were excluded.

The number of patients with a diagnosis of diabetes under the age of 18 and under follow-up in the outpatient clinic is also obtained (data point* B*). The baseline measurement was taken as a ration of number of patients admitted for DKA per total number of patients followed up in clinic* A*/*B*.

Quarterly data of patients 18 years and under who were admitted for DKA was obtained through each ward record. A study team member is allocated on 3-month basis to collect the information and data is compiled in a shared folder used by study team members. A paediatric diabetes clinic database is updated on 3-month basis to include all patients who are 18 years and under with the diagnosis of diabetes and are under regular follow-up in the Diabetes Clinic at the Paediatric Endocrinology Department, Mafraq Hospital.

A yearly ration is calculated with the numerator consisting of number of patients admitted in DKA and denominator of number of patients under regular follow-up at the diabetes clinic.

Patients with diabetes who were admitted acutely for concurrent illnesses or other indications rather than DKA were excluded. Newly diagnosed patients with diabetes presenting with DKA were also excluded. As the measure was specific to our unit performance, patients who presented and were admitted with DKA in our hospital but are not followed up in our outpatient department were excluded too.

### 2.1. Strategy: Setting Up the Diabetes Educator Care Model

Four nurses who are employed to cover diabetes service in the hospital received intensive training to qualify as diabetes educators. Training included provision of a high quality education in all aspects of diabetes management. Educators received special training on the use of technology in treatment of diabetes and were trained on research methodology, data management, and IT related issues through enrollment in various management and IT courses. The educators were 3 females and one male and collectively spoke 6 different languages. The paediatric diabetes case load was divided between the 4 diabetes educators. One diabetes educator was promoted as the paediatric diabetes service coordinator and was in charge of maintaining and updating the clinic database. She also has the task of allocating patients to educators based on

- number of patients per educator,
- preferred gender of educator by family,
- language spoken,
- special interest of the educator in relation to treatment technology used per each patient.

Each patient was allocated a named diabetes educator to be the first line to access of service and was provided with open access to diabetes clinic. Patients were provided with direct contact number of the named educator after hours. Each educator offered 24/7 telephone access to his/her group of patients.

The role of the named educators included introduction of initial and ongoing patient education, setting up group education meetings, training patients at a high level on devices and equipment used to manage diabetes, and conducting awareness campaigns for diabetes. Patients and families were educated on the importance of patient-team collaboration to prevent and treat diabetes complication. They were, also, engaged in various special education programs and involved in departmental surveys and feedback.

Education on DKA recognition, prevention, and management received a high priority in patients and family education. Educators used various audiovisual methods to explain the condition to patients who were given written and digital material for consolidating the knowledge they gain from education sessions. Patients are also trained on using blood and urine ketone strips to detect early signs of DKA. Some patients had access to home blood ketone strips. Regardless of the method of ketone detection, all patients were instructed on the level at which management of ketosis should be started. Detailed instruction on how to avoid and treat DKA has given special attention to patients on insulin pump therapy.

### 2.2. Statistical Analysis

Chi square test is performed to test the difference in admission rate through the study period. *P* value is considered significant if it was found to be less than 0.05.

## 3. Results

### 3.1. Patients and Treatment Characteristics

Majority of patients followed up in clinic had type 1 diabetes. Adolescents with type 2 diabetes constituted around 5% and those with monogenic diabetes (including various types of neonatal diabetes) constituted another 5%. Over the study period, insulin pump use has increased to 50% in 2014 when half of the patients used insulin pumps and the other half was managed on multiple daily injections of insulin.

### 3.2. Diabetes Educators' Work Load and Seasonal Variation

Number of patients per educator increased from around 15 paediatric patients per educator at the start of study to approximately 90 patients in 2014. Diabetes educators received an average of 5 calls per week from patients. More phone calls were received from patients on insulin pumps particularly during the first few days and weeks of pump insertion. The younger the patient was, the more the phone calls were received. There was an obvious increase in phone call consultation over certain seasons of the year. Ramadan fasting month was a season when more phone calls were received.

There were 158 admissions for DKA in children and adolescents 18 years and under over a 5-year period (2009–2014). The number of admissions per year is detailed in Table 1. A total number of patients followed up in the outpatient diabetes clinics ranged between 37–331 patients from 2009 until 2014 (Table 1).

**Figure 1:**
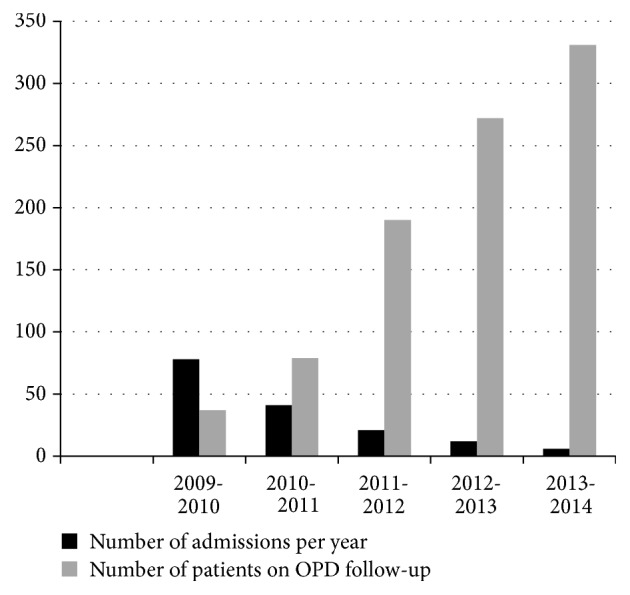


Admission rate showed a downward trend over the five-year period (Figure 1). Ratio between admission episodes and outpatient case load showed a marked reduction from 2.1 in the baseline measurement year to an average of 0.16 over the next 4 years (2010–2014) with a ratio of 0.01 in the last year of the project (2014). Chi square test confirmed that the reduction in the admission rate is statistically significant with a *P* value of 0.001. Admission rate went from a 4.2-fold reduction for the second year after baseline measure to 210-fold reduction 4 years later (*P*  0.001) (Table 1).

## 4. Discussion

DKA is a serious complication of diabetes that can be life threatening. Medical expenditure for this potentially preventable complication is substantial and imposes a large economic burden on the healthcare system [4–7]. Despite substantial progress in overall diabetes management over the past few decades, the incidence of DKA remains high [10].

It is estimated that the risk of DKA in established type 1 diabetes patients can be up to 10% per patient per year [1]. Our study showed that implementation of our diabetes care model has reduced the percentage of DKA admission to 1.8%. Table 1 shows a steady reduction of the DKA admission per year per 100 patients from 210, 51, 11, and 4.4 to 1.8 for years from 2009 to 2014.

DKA is considered potentially entirely preventable complication of type 1 diabetes in young people. Hospital admission because of DKA not only imposes large expenses on health care institute but disrupts the person's routine family life and leads to school and work absence. As the incidence of DKA remains high, its associated hospitalization remains high, too. Curtis et al. showed that over one-third of hospital admissions in children younger than 19 years with type 1 diabetes are due to DKA [15]. Proper education and regular follow-up reduce the possibility of this complication and its resulting hospitalization. Adapting a diabetes educator care model offers patients direct access to support and first-hand troubleshooting advice. Well trained diabetes educator can be the first on-call team members in threating emergencies and can guide patients to proper management to avoid a complication like DKA. Diabetes centers caring for children and adolescents must be equipped with highly trained diabetes educators who can provide a high quality specialized education which will empower patients and families for self-management and provide required support to manage threatening emergencies.

Many barriers are known leading to delay in diagnosing DKA. Of those, there are difficulty in distinguishing cases of DKA from other illnesses, information overload, and ling period from initial diagnosis [16]. Such barriers remain a major obstacle in prevention of DKA in children even in developed countries with active prevention campaigns [17]. Some groups of patients are known to be of high risk to develop DKA and intensive education to prevent this complication should be provided [18, 19].

Our project results proved the importance of training the trainers to deliver the required level of skills and education to the patient (end-user). It showed the importance of establishing a multidisciplinary team in treating a chronic disease like diabetes and minimizing DKA as an acute and possibly life threatening condition. Although training diabetes educators to a high level is expensive, it remains cost-effective when putting the cost of admission for DKA into consideration. 

We had many limitations during execution of the project. Specialist nurses/diabetes educators are not widely available specialists. Employing nurses and training them to the level of diabetes educators incur a high cost and stretch institutions' budgets. Implementing of various patient education programs and preparing unified education material impose time and effort constraints. To accomplish an improvement in the primary end point of reduction hospital admission due to DKA, various devices and expensive ketone-detecting strips are required. These strips utilize capillary blood samples for ketone measurement. They are accurate and can be used reliably instead of plasma or urine samples [20]. Further limitations in prevention of DKA are that some patients are not fully insured and many insurers do not cover diabetes management accessories. In search of diabetes youth study, lack of private health insurance was found to be a risk factor in development of DKA [21]. Implementing and sustaining the diabetes care model can be hard to achieve in institution with limited resources.

There were many limitations to our study. We did not have a control group to confirm effectiveness of the management model. In addition, other factors could contribute to the decrease in DKA, for example, changing the modality of treatment or the insulin regime, increased general improvement of care, and the increase in general awareness of diabetes.

In conclusion, proper guidance on prevention of DKA avoids hospital admission and saves a considerable cost and hospital resources. More importantly, it will empower children and families to self-management and boost their confidence in preventing and treating complications and emergency situation related to diabetes. Our project shows that establishing a diabetes educator care model in managing young people with diabetes is an effective and a sustainable measure to prevent hospital admission of DKA. Out of hours hotline telephone access is a major factor for preventing admission for DKA.

## Additional Points

*Project Summary*. DKA is a serious complication of diabetes that can be life threatening. It is not an uncommon condition with an estimated risk of up to 10% per patient per year. Management of established DKA needs admission in a specialized center and imposes major constraints on hospital resources. Provision of patient education program which includes direct access of care during and after hours can reduce the risk of DKA and minimize the need for hospital admission. We have proposed a model of care led by diabetes educators for children and adolescents with diabetes. The model consists of a package of service for the patient that relies predominantly on structured education and provision of continuity of care and direct access to service. We have measured the effectiveness of the implemented model by comparing the rate of hospital admission for DKA over 4-year period compared to the baseline year prior to implementing the model. We have shown a statistically significant reduction in the admission rate for DKA during the study period. The ratio of the number of admissions to the number of patients followed up in the diabetes outpatient clinic reduced from 2.1 to 0.01 which is equivalent to 210-fold reduction. We conclude that diabetes educator care model is an effective and a sustainable measure way to reduce hospital admission for DKA.

## Figures and Tables

**Table 1:** 

Year	Number of admissions per year (*A*)	Number of patients on OPD follow-up (*B*)	% of admission	Ratio *A*/*B*	Fold reduction compared to baseline
2009-2010	78	37	210%	2.1	Baseline
2010-2011	41	79	51%	0.5	4.2
2011-2012	21	190	11%	0.1	21
2012-2013	12	272	4.4%	0.04	52.5
2013-2014	6	331	1.8%	0.01	210
